# Magnetic Resonance Methods as a Prognostic Tool for the Biorelevant Behavior of Xanthan Tablets

**DOI:** 10.3390/molecules25245871

**Published:** 2020-12-11

**Authors:** Urša Mikac, Julijana Kristl

**Affiliations:** 1 Jožef Stefan Institute, Jamova 39, 1000 Ljubljana, Slovenia; 2 Faculty of Pharmacy, University of Ljubljana, Aškerčeva 7, 1000 Ljubljana, Slovenia; Julijana.Kristl@ffa.uni-lj.si

**Keywords:** hydrophilic matrix tablets, magnetic resonance, hydrogel, drug release, biorelevant dynamic conditions

## Abstract

Hydrophilic matrix tablets with controlled drug release have been used extensively as one of the most successful oral drug delivery systems for optimizing therapeutic efficacy. In this work, magnetic resonance imaging (MRI) is used to study the influence of various pHs and mechanical stresses caused by medium flow (at rest, 80, or 150 mL/min) on swelling and on pentoxifylline release from xanthan (Xan) tablets. Moreover, a bimodal MRI system with simultaneous release testing enables measurements of hydrogel thickness and drug release, both under the same experimental conditions and at the same time. The results show that in water, the hydrogel structure is weaker and less resistant to erosion than the Xan structure in the acid medium. Different hydrogel structures affect drug release with erosion controlled release in water and diffusion controlled release in the acid medium. Mechanical stress simulating gastrointestinal contraction has no effect on the hard hydrogel in the acid medium where the release is independent of the tested stress, while it affects the release from the weak hydrogel in water with faster release under high stress. Our findings suggest that simultaneous MR imaging and drug release from matrix tablets together provide a valuable prognostic tool for prolonged drug delivery design.

## 1. Introduction

Matrix tablets are considered as a dosage form that may maximize the bioavailability of drugs and enable good adjustment of the doses, patient friendly administration, and relatively low manufacturing cost and are, therefore, attractive delivery systems, which are not fully understood yet. The principal objective of dosage form design is to achieve a predictable release and therapeutic response of a drug included in a formulation that is capable of large-scale manufacture with reproducible product quality. The goal of drug administration is to achieve and maintain a plasma drug concentration within the therapeutic window. With conventional oral drug delivery systems, the drug level in the plasma rises after each administration of the tablet and then decreases until the next administration; therefore, frequent dosing is required. However, drug delivery is not easily controlled. In order to avoid the “peaks and valleys” of standard dosage forms, innovative drug delivery systems have been formulated by testing a wide array of hydrophilic polymers and production strategies to prolong drug release and for safe and efficient use. Additionally, matrix tablets with prolonged release are well accepted by patients due to their reduced frequency of administration.

Although the sustained release system was first described in 1952, intensive development is still taking place in this field [1]. Among different types of prolonged drug delivery systems, the most used ones are hydrophilic matrix tablets that are formulated by using a hydrophilic polymer as a material that directs the release kinetics. Basically, these tablets are composed of two major components, a polymer matrix carrier that swells upon exposure to the solvent or physiological environment and the embedded drug molecules that are gradually released [2]. Among hydrophilic polymers, non-ionic polymers of semi-synthetic origin such as hydrophilic derivatives of cellulose ethers [3,4,5] and high molecular weight polyethylene oxides (PEOs) [6] have been extensively studied. Polymers of a natural origin, such as agar–agar, alginate, carrageenan, or xanthan (Xan), are becoming more important in the development of matrix tablets [7,8]. Xan is a well-known and already widely used biopolymer produced by the bacterium Xanthomonas campestris biotechnologically [9]. It is a polysaccharide consisting of a cellulose backbone and trisaccharide side chains containing glucuronic acids and a pyruvate group, which are mainly responsible for its anionic polyelectrolyte character. The native ordered and rigid conformation of Xan chains has been reported to exist as a double-stranded helix [10]. In water, the rigid helix-coil structure transforms into the flexible coils, whose stability and physical properties are strongly influenced by the pH and the ionic environment [7,11]. 

To achieve the optimal performance of the hydrophilic matrix tablets, knowledge of the physicochemical, technological, and physiological parameters that influence the release kinetics is essential for their design [1,12]. One of the key factors for drug release kinetics are the hydrogel properties governed by the hydration of the matrix, mainly defined at the microscopic level by the pore size (i.e., mean linear distance between crossed polymer chains) and their distribution, which are determined by the polymer chain dynamics. These also define the hydrogel swelling kinetics and structure, thus the diffusion of drug through the hydrogel layer [13]. In addition to the hydrodynamic ratio of the drug volume and pore size in the hydrogel, the amount of free water in the network is also responsible for drug diffusion, since the drug has to dissolve before it can diffuse through the hydrogel. Therefore, detailed studies of the amount of free medium within hydrophilic network systems available for drug dissolution are essential to optimize and predict the release kinetics. Methods available for studying the type of water (free water among polymeric chains and water bound to the hydrophilic groups of the polymer) are differential dynamic calorimetry, nuclear magnetic resonance, and Fourier transform infrared spectroscopy [14,15,16,17]. To follow the swelling and the thickness of the hydrogel layers, which also determine drug release, methods such as rheology, gravimetric methods, texture analyzer, optical imaging, ultrasound, microcomputed tomography (micro CT), or magnetic resonance imaging (MRI) are used [4,5,16,18,19,20,21,22,23,24,25,26,27,28]. While the light-based techniques provide high spatial resolution and also enable the spectroscopic characterization of superficial layers of the tablet that are limited by the light penetration depth, MRI enables non-invasive tomographic characterization of the entire hydrogel structure, however with a comparatively larger voxel size. Therefore, experimental studies of tablet swelling that employ various complementary techniques to cover different spatio-temporal scales are commonly combined with a number of different mathematical modelling approaches [29,30,31,32]. On the other hand, also biorelevant conditions may not be neglected. 

Drug formulations administered orally pass through a series of gastrointestinal (GI) compartments with varied contraction forces [33]. It is reasonable to know if the gastrointestinal contraction forces affect hydrogel structure and drug release during GI transit [34]. An approach to distinguish between the role of hydrodynamics and mechanical stresses similar to the contraction forces of the GI tract on drug release from modified release dosage forms was presented by Takieddin’s group [35]. Measuring the effects of these forces in an in vitro setting paves the way for future improvements to drug delivery systems and methods that are more representative of in vivo conditions.

In this work, magnetic resonance imaging (MRI) is used to study hydrogel thickness and drug release from matrix tablets composed of Xan polymer and a non-ionic, highly water soluble drug, pentoxifylline (PF). More precisely, the swelling dynamics of the Xan matrix tablets during hydration and PF release influenced by pH, ionic strength, and mechanical stress caused by medium flow (without flow and with 80 or 150 mL/min flow) are analyzed. To follow the swelling and release under the same experimental conditions and simultaneously, an MRI flow-through system is designed. For these two media differing in pH and ionic strength, the values where the largest differences in Xan swelling and drug release kinetics have been observed in previous studies [18,22] are used: pure water (i.e., with pH 5.7 and ionic strength of 0 M) and an acid medium (i.e., HCl with pH 1.2 and ionic strength of 0.28 M). To show the whole picture of the effectiveness of MR methods in the research on hydrophilic matrix tablets, the results of this study are combined with our previous MR studies of Xan tablets [18,22,36].

## 2. Hydrophilic Matrix Tablets

In their simplest form, hydrophilic matrix tablets are prepared by the compression of a powdered mixture of the drug, a hydrophilic polymer, and other excipients [37]. They do not decompose in contact with body fluids (medium), but a hydrated polymer layer is formed on the surface, which slows the further penetration of the medium and controls the release of the drug. During slow tablet swelling, water diffuses into the tablet, where it is locally taken up by the dry polymer matrix. The medium uptake results in a medium-mediated glassy-to-rubbery phase transition of the polymer matrix. The transition is microscopically manifested by disentangling of individual polymer chains and their cross-linking via medium-mediated intermolecular bridges, while macroscopically, the process is associated with the formation of a hydrogel layer around the tablet core in the glassy state that represents the drug reservoir [38]. The hydrogel layer slows down the penetration of the medium into the tablet and thus the dissolution and diffusion of the drug from the tablet. On the surface of the matrix, the polymer chains disentangle, erode and pass into the surrounding medium.

**Figure 1:**
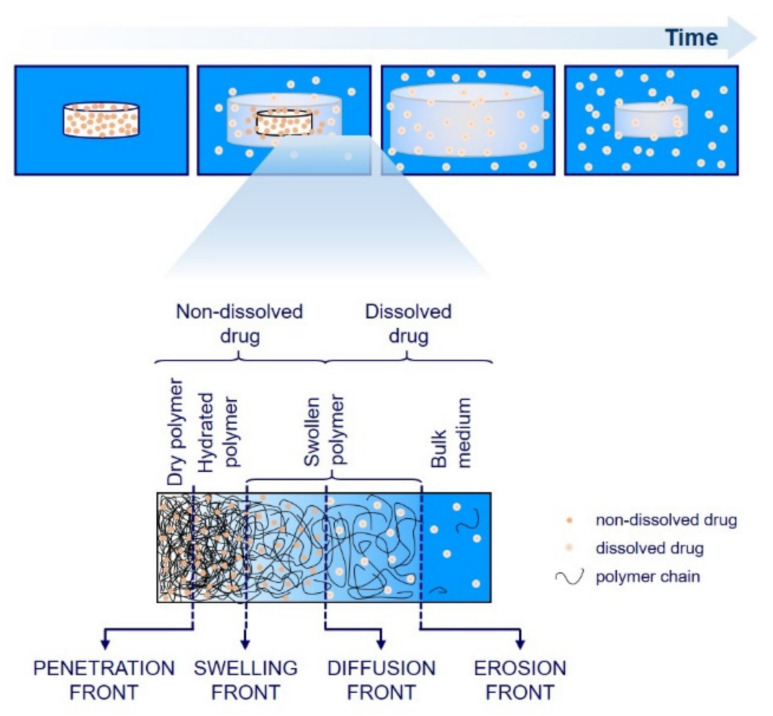
Schematic representation of swelling and drug release from the hydrophilic matrix tablets with different layers and fronts.

The tablet swelling is associated with the formation of four characteristic fronts established in the tablet’s interior during its exposure to the medium [39]. These fronts are: the penetration front that is determined with the maximal reach of the diffusing medium into the tablet interior, i.e., the border between dry and hydrated polymer that is still in a glassy state; the swelling front at the interface between the hydrated glassy polymer and the polymer in a rubbery state (polymer that has taken up a sufficient amount of medium to lower the glass transition temperature *T*g below the experimental temperature); the diffusion front at the interface between undissolved and dissolved drug in the hydrogel layer and the erosion front that contains completely swollen polymer matrix layers in a rubbery state and is in a contact with the bulk medium (Figure 1). As the tablet, immersed in the medium, is subjected to a time-dependent ingress of water molecules into its interior, the positions of these four fronts also become time dependent and determine the efficacy of a controlled drug release. Initially, when the swelling process predominates, the penetration and swelling fronts move toward the center of the tablet and the diffusion and erosion fronts outward. When the concentration of the medium exceeds the critical value, the polymer chains on the hydrogel surface begin to disentangle, and the diffusion and erosion fronts gradually move towards the center of the tablet until the entire tablet disintegrates. 

Drug release from polymer matrix tablets is a very complex process determined by different factors, such as hydrogel layer properties and thickness, polymer-medium and polymer-drug interaction, drug solubility, etc. These factors result in different drug release mechanisms from the hydrophilic matrix tablets [40], such as swelling and erosion controlled release of the drug [1]. In swelling controlled systems, the drug diffuses through the hydrogel layer, while in the erosion controlled systems, the pores in the hydrogel layer are too small to enable drug diffusion. Often, however, the processes of diffusion and erosion occur simultaneously [25].

## 3. Results

**Figure 2:**
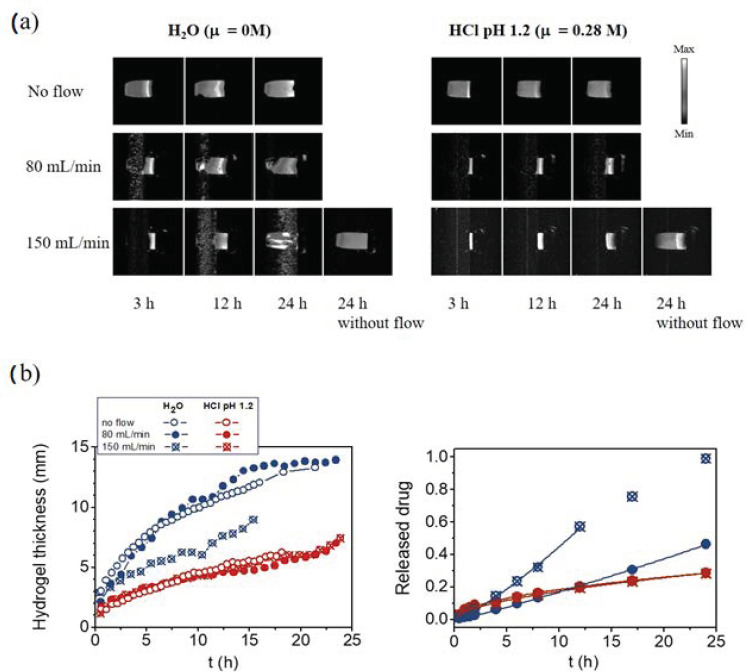
(**a**) 2D MR images of xanthan tablets during swelling at different flow rates in water and in the acid medium and (**b**) corresponding hydrogel layer thicknesses and fractions of the released pentoxifylline drug at different swelling times and mechanical stresses.

The MRI studies using flow-through cells showed some different characteristics of swelling under mechanical stress than in conditions without it. The water profiles in the tablet (polymer matrix) as a function of position and swelling time were obtained through the magnitude of the MRI signal. The signal intensity of 2D MRI depends on the physical state of water in the sample and the chosen experimental conditions. Therefore, it varied through the sample (Figure 2a, upper row). In the MR image, the dry tablet core was black (zero signal), since there was only a small amount of water, and *T*_2_ was too short to give any MRI signal. As the amount of water increased in the hydrogel layer, the signal intensity first increased (from dark grey to white) and, at very high water content, decreased again (from white to grey) due to the long *T*_1_ and short repetition time used in the MRI sequence. Therefore, the brightness of the hydrogel was dependent on the Xan concentration. The signal intensity in the region of pure medium was grey under no flow conditions. The medium flow during MR signal acquisition caused the motional artefacts in the area where only the medium was present (Figure 2a, second and third rows: black regions above the grey hydrogel layer). On the other hand, no artefacts were observed in the hydrogel region. In order to confirm that the MRI signal in the hydrogel region was not affected by the flow, two consecutive images were compared, first without flow and immediately after with the flow. The same hydrogel thicknesses were determined from both images with and without the flow. At the end of the experiment at 150 mL/min flow, it was stopped, and another image without flow was acquired. In water, the MR signal intensity was uniform through the whole sample indicating that after 24 h, no hydrogel layer existed anymore, and disentangled polymer chains were uniformly distributed over the whole cell. The situation was different in acid medium where, after 24 h, the signal intensity still varied through the sample, showing that the hydrogel layer still existed even after 24 h, despite the strong flow.

Moving front positions and the hydrogel thicknesses for Xan tablets swelling in water and in the acid medium at no-flow and at flow rates of 80 mL/min and 150 mL/min were determined from 1D single point imaging (SPI), *T*_2_ values, and 2D images and are shown in Figure 2b. The results show that the thickness of the hydrated hydrogel layers was influenced by the type of medium, flow rate, and swelling time. The hydrated hydrogel layer in water was 1.5 times thicker than in the acid medium. Within the same medium, the flow rate of 80 mL/min did not affect the hydrogel thickness. The situation was different in water at 150 mL/min, where the hydrogel was significantly thinner, and the tablet fully disintegrated at 15 h of swelling. On the other hand, the hydrogel thicknesses were the same for all tested mechanical stresses in the acid medium (Figure 2b).

The drug release profiles at different swelling times were obtained by medium withdrawal during the MRI measurements. Drug release kinetics at both flow rates were compared for Xan tablets swelling in both media (Figure 2b). In water, drug release was significantly faster at higher flow, while the same drug release kinetics were observed at both flow rates in the acid medium. The results of drug release kinetics were compared with Equation (1), and the results of the fitting obtained for each medium are shown in Table 1. In water, the value of the exponent *n* ≥ 1 indicates that drug release was controlled by polymer erosion, and the kinetic constant *k* was higher at a higher flow rate where drug release was faster. The exponent *n* = 0.6 in the acid medium indicates that drug diffusion through the hydrogel layer was the prevailing mechanism for drug release in the acid medium.

Thus, the results indicate that the mechanical stress of the medium flow up to 150 mL/min reduced hydrogel thickness in water and had no effect on the hydrogel thickness in the acid medium, which represents the formation of a remarkably stronger hydrogel in the acid medium. The results agree with the drug release studies, which showed that the release of highly soluble PF drug was significantly increased at higher mechanical forces in water (the rate constant *k* increased from 0.012 h^−1^ at 80 mL/min to 0.025 h^−1.2^ at a flow rate of 150 mL/min) where the main release mechanism was polymer erosion, owing to the weaker hydrogel layer being more susceptible to mechanical forces. In the acid medium, where the main release mechanism was drug diffusion through the hydrogel, the effect of the mechanical stress on the rate constant was negligible.

## 4. Discussion

MR provides information about the hydrogel properties and enables following the moving fronts’ (penetration, swelling, and erosion fronts) positions and the hydrogel’s properties in situ. The spin-spin (*T*_2_) and spin-lattice (*T*_1_) relaxation times measured at the Larmor frequency in the MHz range together with the measurements of the frequency dependent *T*_1_ in the kHz range using the fast field-cycling NMR relaxometry technique provide information about the molecular dynamics over a very wide frequency range. The ability to measure dynamics over a wide frequency range is very important in hydrogels where the molecular dynamics are particularly complicated and can range from the very fast free water dynamics, to slower bound water dynamics, as well as different types of polymer-chain dynamics (fast fluctuations of side groups, different types of backbone motions). The information about medium and polymer dynamics is important for the design of matrix tablets with the desired drug release kinetics since the dynamics determine the diffusion pathways for the drug in the hydrogel layer. By using the MR imaging techniques, the positions of the moving fronts and the hydrogel layer thickness together with polymer concentration profiles across the hydrogel layer during the swelling of the polymer tablets can be determined. MRI experiments using a flow through cell enable simultaneous measurements of the swelling and drug release kinetics, as well as determining the effect of mechanical stress caused by the flow on the hydrogel layer behavior. To better understand the hydrogel impact on drug release, a mathematical model that combines the polymer swelling kinetics and drug release can be applied.

**Figure 3:**
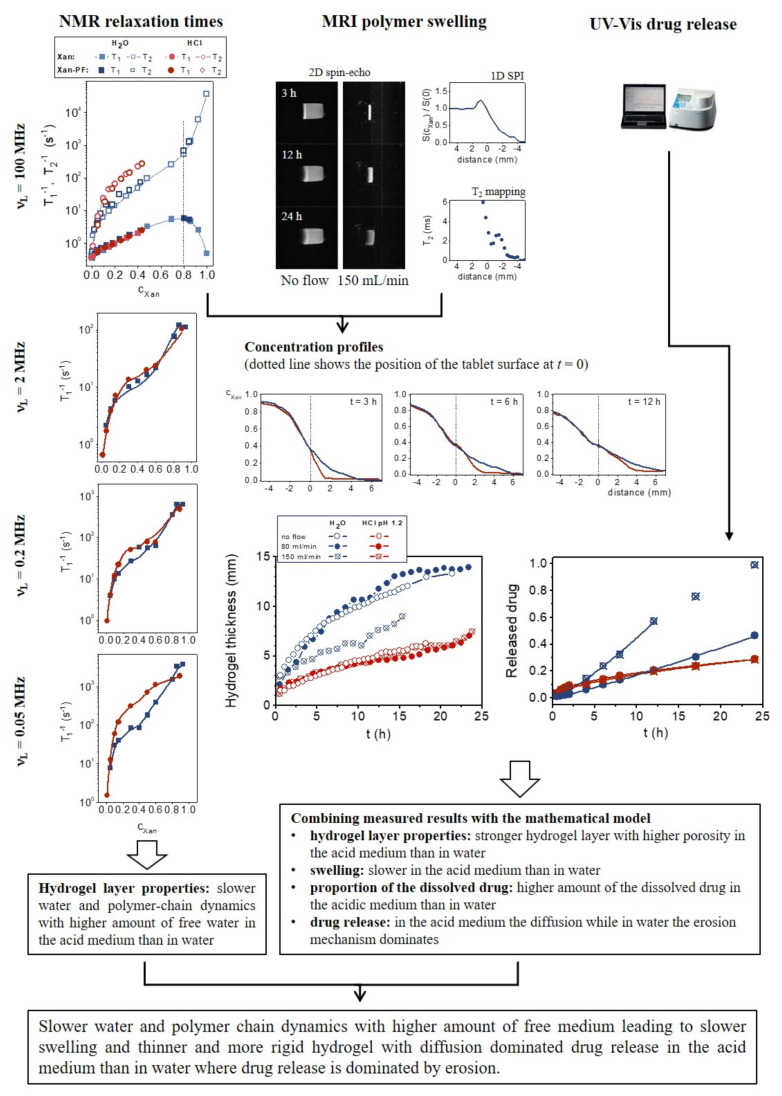
The use of MR methods in the study of the matrix tablets of the xanthan (Xan) polymer.

The ability of MR in the research of hydrophilic matrix tablets and the information that the method can provide are shown in the case of xanthan matrix tablets (Figure 3). Different MR modalities were used to determine and understand the behavior of Xan tablets in media differing in pH and ionic strengths. Besides, the influence of the addition of the highly soluble model drug pentoxifylline on the hydrogel properties was also investigated (Figure 3). Xan is a natural polymer widely used in pharmacy. Due to its polyelectrolyte nature with a pKa of 3.1, its swelling depends on the pH and ionic strength of the medium. In our previous studies, six media that mimic gastric conditions were used [18,22]. It has been shown that the largest differences in Xan swelling and drug release are between a medium with pH 1.2 and pH 3.0; between pH 3.0 and water with pH 5.7, the differences are extremely small. No differences were observed between water and the medium with pH 7.4, so this medium was not included in further investigation with Xan. Therefore, the results of Xan where the largest differences were observed, HCl medium with pH 1.2 and ionic strength µ = 0.28 M and purified water with pH 5.7 and µ = 0 M, will be discussed. By measuring the spin-spin and spin-lattice relaxation times of hydrogels with a known Xan concentration, we found that the high frequency dynamics (measured by *T*_1_ at 100 MHz) were the same in both media, while the dynamics at low frequencies depended on the medium properties [18,36]. The effect of medium pH and ionic strength resulted in slower medium and polymer-chain dynamics with a higher amount of free water available for drug dissolution in the hydrogels prepared with the acid medium than in the hydrogels prepared with water. No impact on the dynamics was observed after the addition of the PF drug in Xan hydrogels with a Xan to PF ratio of 1:1. To determine how the medium properties affect the swelling kinetics of the Xan tablets, MR imaging was used. By knowing how the NMR relaxations change with the polymer concentrations (concentration dependencies of *T*_1_ and *T*_2_ measured at 100 MHz), the MR imaging parameters (*TE* and *TR*), which provide the best contrast between the bulk medium, hydrogel, and dry tablet, can be determined. In addition, the polymer concentration profiles across the formed hydrogel can also be calculated for different swelling times. MRI showed that the pH and ionic strength of the media significantly influenced hydrogel layer thickness, i.e., by decreasing the pH or increasing the ionic strength of the medium, the hydrogel layer thickness decreased. Different hydrogel layer thicknesses were the result of different erosion front positions, while the positions of the penetration and swelling fronts were independent of the medium properties [18,22]. These can also be seen from the concentration profiles where the concentration of Xan in the acid medium decreased much faster at low polymer concentrations than in water (Figure 3). The influence of the mechanical stress on the hydrogel layer formation showed that the hydrogel layer was weaker in water, where the hydrogel layer was significantly thinner at a high flow rate, and the tablet disintegrated after 15 h (e.g., the hydrogel thickness was 7.4 mm at 80 mL/min and 5.3 mm at a 150 mL/min flow rate after 5 h and 10.7 mm at 80 mL/min and 6 mm at a 150 mL/min flow rate after 10 h of swelling), than in the acid medium where the flow up to 150 mL/min did not affect the hydrogel layer (e.g., the hydrogel thickness was 3.4 mm after 5 h and 4.4 mm after 10 h of swelling for both flow rates). The addition of PF drug in the Xan tablets showed that at a high drug amount (Xan:PF = 1:1), the hydrogel layer was thinner in media with a low pH or increased ionic strength than in the empty Xan tablets with no PF influence observed in water [22].

By using a mathematical model, which combines the polymer swelling kinetics and drug release that account for the superposition of Fickian diffusion and polymer erosion processes, the results of Xan swelling and the PF release kinetics can be better understood [22]. The obtained model parameters showed that in water, the fraction of dissolved drug was lower, and the main release mechanism was erosion; whereas in the acid medium, the amount of medium in the tablet that was available for PF dissolution was higher, and the main mechanism was drug diffusion. The parameters also clarify the reason for the unexpected behavior; i.e., the same hydrogel thickness and slightly faster drug release in water, on the one hand, and the thinner hydrogel and the same drug release kinetics in the acid medium for high (Xan:PF = 1:1) compared to low (Xan:PF = 3:1) drug loading, on the other hand. In water, more medium was available for drug dissolution and, consequently, for drug release in tablets with high drug loading, causing faster drug release despite the same hydrogel layer thickness. In acid medium, the smaller diffusion contribution led to a thinner hydrogel in the tablets with high drug loading; despite the slower drug diffusion, drug release was the same for both drug loadings owing to the higher amount of dissolved drug and the thinner hydrogel layer in tablets with high drug loading compared to the tablets with low drug loading.

Different MR measurements thus showed that more restricted mobility in the acid medium resulted in a thinner hydrogel layer, which was more resistant to erosion, with the drug release mainly governed by drug diffusion through the hydrogel layer. In water, the higher water and polymer-chain mobility resulted in a weaker and homogeneous hydrogel layer that was less resistant to erosion, leading to erosion controlled drug release. At low mechanical stress, the release was faster in the case of the diffusion controlled mechanism (acid medium) than in the erosion mechanism (water): at an 80 mL/min flow rate, the fraction of released drug was 0.06 in water and 0.11 in the acid medium after 4 h and 0.13 in water and 0.16 in the acid medium after 8 h of swelling. At longer times when the hydrogel was more diluted and, consequently, weaker, the erosion controlled release in water became faster than the diffusion controlled release in the acid medium (after 24 h, the fraction of released drug was 0.46 in water and 0.28 in the acid medium). When strong mechanical forces were applied, causing more pronounced hydrogel erosion in the weaker hydrogel in water, drug release was accelerated. This led to faster drug release in water than in the acid medium (e.g., at a 150 mL/min flow rate, the fraction of released drug was 0.15 in water and 0.11 in the acid medium after 4 h of swelling and 0.32 in water and 0.16 in the acid medium after 8 h of swelling), where the hydrogel was so robust that the flow up to 150 mL/min did not affect the hydrogel layer, and the main release mechanism remained drug diffusion.

## 5. Materials and Methods

### 5.1. Materials

Xanthan with a MW of 2 × 10^6^ was obtained from Sigma-Aldrich Chemie, Munich, Germany. A model drug, pentoxifylline (PF) (MW = 278.31) with a solubility in water at 25 °C of 77 mg/mL, was supplied by Krka, d.d. Novo mesto, Slovenia. For swelling and release experiments, two different media were used: purified H_2_O with pH 5.7 and ionic strength of 0 M and HCl at pH 1.2 with increased ionic strength (11.7 g of NaCl per 1000 mL of HCl medium) resulted in ionic strength µ = 0.28 M.

### 5.2. Preparation of Xanthan Matrix Tablets

Xan and the drug (PF) were mixed homogeneously using a laboratory model drum blender (Electric Inversina Tumbler Mixer, Paul Schatz principle, BioComponents Inversina 2L, Bioengineering AG, Wald, Switzerland), and cylindrical flat-faced tablets with composition of Xan:PF = 1:1 (200 mg of Xan and 200 mg of PF) were prepared by direct compression (SP 300, Kilian and Co., Cologne, Germany) to form tablets with a diameter of 12 mm and a crushing strength of 100 N ± 10 N (Tablet hardness tester, Vanderkamp, VK 200, USA).

### 5.3. MRI of Xanthan Tablets during Swelling

The MRI experiments were performed at room temperature with a superconducting 2.35 T (^1^H NMR frequency of 100 MHz) horizontal bore magnet (Oxford Instruments, Oxon, U.K.) equipped with gradients and RF coils for MR macroscopy (Bruker, Ettlingen, Germany) using a TecMag Apollo (Tecmag, Houston, TX, USA) MRI spectrometer.

To follow the swelling and release of the drug from Xan tablets under the same experimental conditions and simultaneously, a flow-through system was designed. The tablet was inserted in a small MRI flow-through cell so that only one circular cylinder surface was exposed for the medium penetration. The flow-through cell was connected with the container with 900 mL of medium using plastic tubes. To determine drug release from the same Xan tablets and at the same time as MRI, five milliliter samples were withdrawn at predetermined time intervals. This means that the MRI scan and dissolution medium withdrawing were performed simultaneously. Gastrointestinal tract (GIT) mechanical stress was simulated with different flow rates of the dissolution medium, which was driven by a peristaltic pump (Anko, Bradenton, FL, USA) and controlled at two different flow rates: 80 ± 2 mL/min and 150 ± 2 mL/min. To determine the influence of the stress on hydrogel thickness and drug release, the experiments were also performed without flow. The first MR image was taken approximately 10 min after the tablet came in contact with the medium and then every 30 min for 24 h. The experiments were performed at room temperature (≈22 °C).

**Figure 4:**
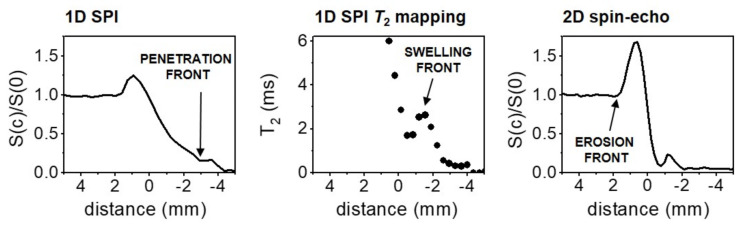
Determination of the moving fronts: the penetration front was determined from 1D SPI normalized signal intensity; the swelling front was determined from the *T*_2_ profile; and the erosion front was obtained from the one-dimensional signal intensity profile along the horizontal direction of the 2D MR image. Zero on the x axis represents the surface of the tablet at the beginning of the experiment.

To follow the moving (penetration, swelling, and erosion) front positions, two different MRI methods were used as described in our previous paper [18]: a 2D multi-echo pulse sequence to determine the erosion front and a 1D SPI *T*_2_ mapping sequence to determine the position of the swelling and penetration fronts. Imaging parameters for the 2D multi-echo pulse sequence were: an echo time (*TE*) of 6.2 ms, a repetition time (*TR*) of 200 ms, a field of view of 50 mm with an in-plane resolution of 200 µm, and a slice thickness of 3 mm. For the 1D SPI, a single point on the free induction decay was sampled at the encoding time *t*_p_ of 0.17 ms after the radiofrequency detection pulse α of 20° with *TR* = 200 ms, and the inter-echo time was varied from 0.3 ms to 10 ms. The field of view was 45 mm with an in-plane resolution of 350 µm. The position of the erosion front was obtained from the one-dimensional signal intensity profiles of the 2D MR images; the position of the swelling front was determined from 1D SPI *T*_2_ maps at *T*_2_ = 2.7 ms; and the position of the penetration front was determined from the signal intensity profiles from SPI measurements at an inter-echo time of 0.3 ms (Figure 4).

### 5.4. Drug Release from Xanthan Tablets

To determine drug release from the Xan tablets, the collected samples of the outflow medium from the MRI flow-through cell were filtered through a filter with 0.45 μm pores. Drug release was monitored as a function of time using the HP Agilent 8453 Diode Array UV-Vis Spectrophotometer, Waldbronn, Germany, to measure the absorbance of PF at 274 nm.

Drug release profiles were analyzed using the Korsmeyer–Peppas equation [41,42]:

(1) $$\frac{M\;\left(t\right)}{M\;\left(\infty \right)}=k\;\cdot \;t^{n},$$

where *M*(*t*)/*M*(∞) is the fraction of the released drug at time *t*, *k* is the rate constant, and *n* is the diffusion exponent, which indicates the general release mechanism: *n* = 0.5 indicates Fickian diffusion controlled drug release (Case I), and *n* = 1.0 indicates Case II transport (erosion controlled drug release). Case I release occurs by molecular diffusion of the drug due to a chemical potential gradient, whereas the mechanism driving Case II drug release is the swelling or relaxation of polymeric chains. Values between 0.5 < *n* < 1.0 indicate an anomalous (non-Fickian or both diffusion/erosion) controlled drug release. Equation (1) is the short time approximation, which is valid only up to the fraction of 0.6 of released drug.

## 6. Conclusions

NMR can be used to determine the structure of the hydrogel layer and MR imaging as a non-destructive and fast enough method, which allows monitoring the swelling of hydrophilic matrix tablets in situ. Here, matrix tablets composed of xanthan polymer and a non-ionic, highly water soluble drug pentoxifylline are investigated using the MRI flow-through cell with simultaneous drug release testing. Inclusion of in vitro mechanical stress simulating GI contraction forces during dissolution testing allows for better in vivo prediction.

The swelling dynamics of the Xan matrix tablets during hydration and PF release influenced by the medium properties and mechanical stress caused by medium flow (without flow and with 80 or 150 mL/min flow) were analyzed. The results of this study together with the previous MR studies of Xan tablets show the more restricted mobility of Xan polymer chains and water molecules in the acid medium than in water, which results in a thinner hydrogel layer more resistant to erosion. Xan matrix in the acid medium releases PF mainly by drug diffusion through the strong hydrogel layer. Xan in water swells faster due to the higher water and polymer-chain mobility, resulting in a weaker hydrogel, less resistant to erosion, which causes the erosion controlled drug release. High mechanical stress affects the release from the soft hydrogel in water, while the release of the harder hydrogel in the acid medium remains the same, independent of mechanical stress. 

The combination of the obtained results together with the results of other analytical methods and applied mathematical models enables the understanding of polymer systems at the molecular, microscopic, and macroscopic levels and therefore contributes to the development of efficient systems with the desired drug release kinetics.

## Figures and Tables

**Table 1:** Values of the fitting parameters determined from drug release data measured at two different flow rates and fit by Equation (1) in water and in the acid medium.

	H_2_O (μ = 0 M)		HCl pH 1.2 (μ = 0.28 M)	
Flow Rate	*k* (h^−n^)	*n*	*k* (h^−n^)	*n*
80 mL/min	0.012	1	0.055	0.6
150 mL/min	0.025	1.2	0.048	0.6
